# Combination treatment with hENT1 and miR-143 reverses gemcitabine resistance in triple-negative breast cancer

**DOI:** 10.1186/s12935-022-02681-0

**Published:** 2022-09-01

**Authors:** Yue Xi, Ting Li, Yun Xi, Xinyi Zeng, Ying Miao, Rui Guo, Min Zhang, Biao Li

**Affiliations:** 1 Department of Nuclear Medicine, Ruijin Hospital, Shanghai Jiao Tong University School of Medicine, 197 Ruijin 2nd Road, Shanghai, 200025 China; 2 Collaboration Innovation Center for Molecular Imaging of Precision Medicine, Ruijin Center, Shanghai, 200025 China; 3 Shanghai Institute of Hematology, State Key Laboratory of Medical Genomics, National Research Center for Translational Medicine at Shanghai, Ruijin Hospital, Shanghai Jiao Tong University School of Medicine, Shanghai, China

**Keywords:** Triple-negative breast cancer, hENT1, miR-143, Combination therapy, Gemcitabine resistance

## Abstract

**Background:**

Triple-negative breast cancer (TNBC) is an aggressive subtype of breast cancer and is susceptible to develop gemcitabine (GEM) resistance. Decreased expression of human equilibrative nucleoside transporter 1 (hENT1) accompanied by compensatory increase of glycolysis is strongly associated with GEM resistance in TNBC. In this study, we investigated the treatment feasibility of combined hENT1 upregulation and miR-143-mediated inhibition of glycolysis for reversing GEM resistance in TNBC.

**Methods:**

Experiments were performed in vitro and in vivo to compare the efficacy of GEM therapies. In this study, we established stable drug-resistant cell line, GEM-R cells, from parental cells (MDA-MB-231) through exposure to GEM following a stepwise incremental dosing strategy. Then GEM-R cells were transfected by lentiviral plasmids and GEM-R cells overexpressing hENT1 (GEM-R-hENT1) were established. The viability and apoptosis of wild-type (MDA-MB-231), GEM-R, and GEM-R-hENT1 cells treated with GEM or GEM + miR-143 were analyzed by CCK8 assay and flow cytometry. The RNA expression and protein expression were measured by RT-PCR and western blotting respectively. GEM uptake was determined by multiple reaction monitoring (MRM) analysis. Glycolysis was measured by glucose assay and ^18^F-FDG uptake. The antitumor effect was assessed in vivo in a tumor xenograft model by evaluating toxicity, tumor volume, and maximum standardized uptake value in ^18^F-FDG PET. Immunohistochemistry and fluorescence photography were taken in tumor samples. Pairwise comparisons were performed using Student’s t-test.

**Results:**

Our results represented that overexpression of hENT1 reversed GEM resistance in GEM-R cells by showing lower IC_50_ and higher rate of apoptosis. MiR-143 suppressed glycolysis in GEM-R cells and enhanced the effect of reversing GEM resistance in GEM-R-hENT1 cells. The therapeutic efficacy was validated using a xenograft mouse model. Combination treatment decreased tumor growth rate and maximum standardized uptake value in ^18^F-FDG PET more effectively.

**Conclusions:**

Combined therapy of exogenous upregulation of hENT1 expression and miR-143 mimic administration was effective in reversing GEM resistance, providing a promising strategy for treating GEM-resistant TNBC.

## Background

Breast cancer is a global health challenge and the leading cancer-related cause of disease burden among women [1–4], and disease incidence is increasing [5–7]. Of all types of breast cancer, triple-negative breast cancer (TNBC) is the most aggressive and lethal subtype, affecting 12–18% of breast cancer patients [8], and is characterized by the lack of expression of estrogen receptors, progesterone receptors, and human epidermal growth factor receptor 2 (HER2); for this reason, endocrine or anti-HER2 targeted therapies are not effective [9]. TNBC is more prevalent in younger women and challenging to treat because of a higher rate of relapse and metastasis and a lower survival rate than other types of breast cancer [10, 11]. Since no effective targeted therapies are currently available to improve survival in patients with TNBC because the molecular mechanisms of recurrence are unknown, chemotherapy remains the mainstay of treatment [12]. However, TNBC tends to develop drug resistance, so that affected patients have a higher risk of recurrence and worse prognosis after chemotherapy [13].

Gemcitabine (GEM), an antimetabolite antineoplastic drug [14] that functions as a nucleoside analog and inhibits DNA replication, is useful in the treatment of pancreatic cancer, metastatic breast cancer and so on [15, 16]. GEM is the first-line treatment for metastatic TNBC with or without paclitaxel or other drugs [17–19]. Nevertheless, tumor resistance to GEM increases the risk of relapse and decreases survival and the quality of life [8, 20, 21]. Therefore, reducing tumor resistance to chemotherapy is essential to improve survival and the quality of life of patients with TNBC.

The biochemical mechanisms of drug resistance include [22] reduced drug delivery, uptake, and metabolism, and increased drug efflux and inactivation. Although the molecular mechanisms of drug resistance are incompletely understood, evidence has indicated that transporter proteins play an important role in the uptake and efficacy of chemotherapeutic agents and drug resistance [23, 24]. Human equilibrative nucleoside transporter 1 (hENT1) is a member of nucleoside transmembrane transporter proteins and mediates the cellular uptake of nucleoside drugs such as GEM [25, 26]. Previous studies have demonstrated that hENT1-deficient cells are less sensitive to nucleosides in vitro [27–29]. hENT1 is found downregulated after drug resistance develops in multiple cancers, including pancreatic cancer [30], cholangiocarcinoma [31], and breast cancer [27]. Moreover, the expression level of hENT1 is strongly associated with prognosis and the efficacy of GEM-based therapies in pancreatic [32, 33], lung [34], and breast cancer [27]. These data suggest that upregulating the expression of hENT1 is a potentially effective strategy for reversing GEM resistance and enhancing treatment efficacy in TNBC.

The enhancement of glycolysis is considered to be a compensatory mechanism for tumor cells by downregulating the expression of adenosine transporters to reduce the entry of gemcitabine drugs and adenosine energy substances into the cell [35, 36], and to participate in the resistance of tumor cells to GEM. MicroRNAs (miRNAs) are small endogenous non-coding RNAs that reduce the expression and stability of mRNAs [37, 38]. *MiR-143* inhibits glycolysis [39] in glioblastoma [40], gallbladder cancer [41], and breast cancer [42], suggesting its potential as an oncomiR [43, 44], and is markedly downregulated in TNBC. Moreover, *miR-143* is associated with tumor invasiveness, metastasis, and chemoresistance [45, 46]. *MiR-143* inhibits [44] glycolysis by downregulating hexokinase 2 (HK2), the rate-limiting enzyme in the glycolytic pathway. Therefore, *miR-143* can potentially reduce the Warburg effect [47] and reverse GEM resistance in TNBC.

This study proposed a combination therapy involving the exogenous overexpression of hENT1 and the administration of *miR-143* mimic to increase GEM uptake and decrease glycolysis in tumor cells, and assessed the efficacy of this therapy to reduce GEM resistance in vitro and in an animal model of TNBC.

## Methods

### Patient survival analysis

The distal recurrence free survival data of TNBC patients from GSE25066 and GSE69031 was obtained from Gene Expression Omnibus (GEO, http://www.ncbi.nlm.nih.gov/geo/) [48] Kaplan–Meier analysis was performed to determine the overall survival of TNBC patients using the online database (http://kmplot.com/analysis/), [49, 50]. The array probe of hENT1(SLC29A1) was 201801_s_at. We selected TNBC patients with ER, PR and HER2 negative status from both array and immunohistochemistry (IHC). The best cut-off was applied.

Patient survival was evaluated with Kaplan–Meier method and log-rank test was used to establish the statistical significance of the distance between curves by Statistical Product and Service Solutions (SPSS) v25.0 and GraphPad Prism v6.0 software.

### Cell culture

The human TNBC cell line MDA-MB-231 (wild-type [WT]) was purchased from Cell Bank of the Chinese Academy of Sciences, Shanghai, China. The cell line passed the library mycoplasma test and the results were negative. It has been authenticated by STR. Cells were cultured in DMEM (Gibco, NYS, USA) which was added with 4.5 g/L glucose, 10% fetal bovine serum (FBS) (Gibco, NYS, USA), and 1% penicillin–streptomycin (PS) (Sigma, St Louis, MO, USA) at 37 °C in a humidified incubator with 5% CO_2_.

### Establishment of stable GEM-R and GEM-R-hENT1 cell lines

Gemcitabine-resistant MDA-MB-231 (GEM-R) cells and GEM-R cells with exogenous expression of the hENT1 gene (GEM-R-hENT1) were established as described in our previous study [51].

GEM-R cells were obtained by culturing WT cells in DMEM containing GEM for 24 h, followed by culturing in fresh medium without GEM for about one-week recovery until stable proliferation was observed. For the GEM exposure, stepwise incremental doses from 1 to 20 μM were applied. The development was repeated for at least two cycles (Fig. 2A).

To obtain GEM-R-hENT1 cells, recombinant lentivirus plasmids LV-EF1α-IRES-puro and LV-EF1α-hENT1-IRES-puro were constructed by Shanghai Xitubio Biotechnology Co., Ltd. hENT1 amplification was confirmed by RT-PCR. For transfection, GEM-R cells were plated in 6-well plates (2 × 10^5^ cells per well) and cultured in DMEM for 24 h. Culture medium containing 8 μg/mL polybrene (SiDanSai Biotechnology Co., Ltd, Shanghai, China) was mixed with lentiviral supernatant at a ratio of 1:1 and added to each well, and the medium was changed after 24 h incubation. Cells stably expressing hENT1 were screened with 2 μg/mL puromycin (Sangon Biotech, Shanghai, China).

### MiRNA transfection

Cells were transfected with MicrON™ *has-miR-143-3p* mimic (RiboBio, Guangzhou, China) using Lipofectamine 2000 (Invitrogen, MA, USA) according to the manufacturer’s protocol. After cell seeding into plates, Lipofectamine 2000 reagent was diluted in Opti-MEM (medium A) and micrON™ *has-miR-143-3p* mimic was diluted in Opti-MEM medium (medium B) respectively (for different samples, the dilution rate varies). Then, diluted medium A and B were added at a ratio of 1:1 to incubate for 5 min at room temperature to get the RNA-lipid complex. Next, the RNA-lipid complex was added to each well to get cells transfected.

### Cell counting kit-8 (CCK-8) assays

The IC_50_ of all cell lineages was measured. Approximately 6 × 10^3^ cells per well were seeded in 96-well plates. Then, a gradient concentration of GEM from 0 to 2 mM was added to the culture medium, and cells were grown for 72 h. Cytotoxicity was evaluated using the CCK-8 assay (Beyotime Biotechnology, Shanghai, China) according to the manufacturer’s instructions. After 2 h of the addition of CCK8, the absorbance value was measured at 450 nm with an enzyme-labeled-meter (Thermo Fisher Scientific, multiskan MK3). IC_50_ was defined as the GEM concentration that killed 50% of cells. All experiments were performed in triplicate and repeated independently three times.

The viability of MDA-MB-231 and GEM-R cells was determined. Cells were seeded in 96-well plates at a density of 6,000 per well (100 μL) and cultured in DMEM with and without glucose. After 72 h incubation, CCK8 assay was performed as described above.

### Flow cytometry for apoptosis detection

Apoptosis was measured by a flow cytometry detector (Beckman Cytoflex S) using the Annexin V-FITC Apoptosis Detection kit (Sigma, St Louis, MO, USA) following the manufacturer’s instructions.

Cells are seeded in 6-well plates at a density of 1 × 10^5^/ml. After 72 h of dosing treatment, the upper medium (v1) of each well is collected separately in flow cytometry tubes (flow cytometry tubes are labeled according to the sample wells). Cells are then washed twice with 1 × PBS buffer, and the wash solution (v2) is also collected separately in new flow cytometry cell tubes. 100 μL of EDTA-free trypsin (Beyotime Biotechnology, Shanghai, China) was added to each well to completely cover the cells at the bottom of the wells. After about 1–2 min’ digestion, v1 medium was added to corresponding well to terminate digestion.

Next, cells were centrifuged at 500*g*, 4 °C in an ultracentrifuge to get cell suspension. Then cells were washed twice with 1 × PBS buffer. Cells were resuspended in 200 μl Binding Buffer (1 ×). 5 μl FITC was added into the cell suspension, well mixed and incubated for 10 min at room temperature. Cells were washed with 200 μl binding buffer (1 ×) and resuspended in 95 μl binding buffer (1 ×). Before detection, 5 μl of propidine iodide was added to cell suspension and apoptosis was detected by a cell flow cytometry detector (Beckman Cytoflex S).

### Real-time PCR

The expression levels of hENT1, miR-143, and HK2 were determined by real-time PCR on a ViiA™ 7 Real-Time PCR System (Applied Biosystems). RNA was extracted using the RNeasy Mini Kit (Qiagen, Germany) and reverse-transcribed into cDNA using the PrimeScript RT Master Mix (TAKARA, Japan). The primer sequences are listed in Table 1. Amplification conditions included a denaturation step at 95 °C for 30 s, followed by 40 cycles at 95 °C for 5 s, 60 °C for 30 s, and 72 °C for 5 min. Relative mRNA expression was evaluated using the 2^−ΔΔCt^ method (β-actin as endogenous reference).

**Table 1:** Primer sequences for Real-time PCR

Name	Forward	Reverse
Primer sequences		
hENT1	5ʹ-CTGGCTTTCTCTGTCTGCTTCA-3ʹ	5ʹ -CTCAACAGTCACGGCTGGAA-3ʹ
miR-143	5′-ACACTCCAGCTGGGTGAGATGAAGCACTGT-3′	5ʹ-CTCAACTGGTGTCGTGGAGTCGG-3ʹ
HK2	5′-AAGGCTTCAAGGCATCTG-3′	5ʹ-AAGGCTTCAAGGCATCTG-3′

### Western blotting

Proteins were extracted from GEM-R and GEM-R-hENT1 cells using RIPA buffer (Sigma, St Louis, MO, USA), and protein concentration was determined using the BCA Protein Assay Kit (Beyotime Biotechnology, Shanghai, China). 7.5% PAGE Gel Fast Preparation Kit (Epizyme, Shanghai, China) was used to prepare gels. Protein samples were mixed with 5 × loading buffer from Epizyme at a volume ratio of 4:1, boiled at 100℃ for 10 min, electrophoresed, and transferred to a polyvinylidene fluoride membrane (Sangon Biotech, Shanghai, China). The membrane was blocked in protein-free rapid blocking buffer (1 ×) for 10 min and incubated with a primary antibody against ENT1 (Abcam, UK; Table 2) and β-actin at dilutions of 1:1000 at 4° overnight. The membrane was washed with TBST three times and incubated with HRP-labeled anti-rabbit/mouse secondary antibody (Abcam, UK; Table 2) for 1 h at room temperature. Bands were visualized using the ChemiDoc XRS system (BioRad) and quantified using ImageJ version 1.5. Grayscale value ratio = The area of the target protein band/The area of the endogenous reference protein band. For GEM-R cells treated with and without *miR-143*, primary antibodies against HK2 and β-actin (Abcam, UK; Table 2) at dilutions of 1:1000 were used.

**Table 2:** Antibodies for Western blot and immunohistochemistry

Name	Antibody type	Host	Target species	Purification	Experiment	Company
hENT1	Primary	Rabbit	hENT1	polyclonal	WB/IHC	Abcam
HK2	Primary	Rabbit	HK2	monoclonal	WB/IHC	Abcam
β-Actin	Primary	Mouse	β-Actin	monoclonal	WB	Abcam
Goat anti-rabbit HRP	Secondary	Goat	Rabbit IgG	/	WB/IHC	Abcam
Goat anti-mouse HRP	Secondary	Goat	Mouse IgG	/	WB/IHC	Abcam

*WB* western blot, *IHC* immunohistochemistry

### Multiple reaction monitoring (MRM)analysis and GEM uptake determination

The concentration of GEM was quantified using a HPLC System (LC-30A, SHIMADZU, JAPAN) coupled with a triple quadrupole mass spectrometer (QTRAP6500, SCIEX, USA) for MRM analysis (264.1/112.1). Capillary voltage was set at 4500 V. MRM Declustering Potential (DP), Entrance Potential (EP), Collision Energy (CE), Collision Cell Exit Potential (CXP) were optimized for each metabolite by FIA mode. The detection was operated in positive mode. A total of 2 μL sample was separated on an Accucore™ C18 column (2.1 × 100 mm) with solvent A (95% H_2_O/5% Acetonitrile [ACN]/0.1%Formic acid [FA]) and solvent B (95% ACN/%H_2_O/0.1%FA). The flow rate was set at 0.3 mL/min and the column temperature was kept at 40 °C. Elution was completed by holding 90% A and 10% B for 0.3 min, then a linear gradient to 90% B and 10% A within 4 min and the system returned to the initial conditions within 30 s.

WT, GEM-R and GEM-R-hENT1 cells were cultured in cell culture dishes and incubated in a cell culture incubator. When cells were grown to the confluent of 70%–80%, 10 µM GEM was added to each cell dish and mixed thoroughly with the medium. For the GEM uptake determination, each cell sample (about 3 × 10^6^ cells) was added with 1 ml 50% methanol water solution. After repeated freezing and thawing in liquid nitrogen, the cells were completely broken and were centrifuged at 13200 rpm for 20 min at 4 ℃. The supernatants were lyophilized and redissolved in methanol /acetonitrile/ water solution (2:2:1). The mixture was vortexed until all the precipitates were dissolved and then centrifuged again. The supernatants were added to the loading bottle for detection.

The GEM standard curve was established with 19 standard solutions in the range between 10 and 1000 ng/mL (r^2^ > 0.99). The amount of GEM uptake of WT, GEM-R and GEM-R-hENT1 cells was determined by calculating the area under the elution peak and the area of ​​the standard curve.

### Measurement of glucose consumption and in vitro ^18^F-FDG uptake

WT, GEM-R, and miR-143-treated GEM-R cells were cultured in glucose-free DMEM in 24-well plates for 2 h at cell density of 1 × 10^5^/ml. Then for each well, cells were added with five microliters of DMEM containing 4.5 g/L glucose and were grown for another 1 h. A glucose assay kit (Sigma) was utilized to measure glucose concentration in the medium. To measure ^18^F-FDG uptake, cells were grown in 24-well plates for 24 h in a cell culture incubator, washed three times with PBS, and cultured in 200 μL of glucose-free medium containing 10 μM GEM. Experiments were carried out in triplicate. After 2 h incubation, 37 kBq/mL ^18^F-FDG was added to the medium, and cells were cultured for 1 h. The medium was aspirated into tubes for later testing. Ice-cold PBS was used for washing cells for three times and then was mixed with the medium as supernatants. Cells were digested with 0.25% trypsin and collected into another tube. Cold PBS was used for washing wells and was mixed with the cells. ^18^F-FDG radioactivity was measured in cells (B) and supernatants (F) by a gamma counter, and in vitro ^18^F-FDG uptake rate (%) was calculated according to the formula as previously described [52]: uptake rate (%) = [B / (B + F)] × 100%.

### *Xenograft model and treatment experiments *in vivo

All animal experiments conformed to the guidelines of the Animal Care and Use Committee of Ruijin Hospital, School of Medicine of Shanghai Jiao Tong University, and to the National Institutes of Health Guide for the Care and Use of Laboratory Animals (Publication No. 8023, revised in 1978).

Five-week-old immunodeficient female BALB/c nude mice (Lingchang Animal Experiment Center) served as a subcutaneous xenograft model. MDA-MB-231, GEM-R, and GEM-R-hENT1 cells were injected subcutaneously into mice to establish three tumor models. Group allocation is shown in Tables 3, 4, 5. Gemcitabine dissolved in 0.9%NaCl solution (20 mg/kg) was injected intraperitoneally every 5 days for eight cycles. Mice treated with miR-143 were injected with 2 nmol of miRNA agomir into the tumor site every 3 days for eight cycles (Fig. 3A). The tumor volume growth curve was analyzed by measuring tumor length (L) and width (W) every 2 days and tumor volume using the formula (LW2)/2. Body weight was measured every 2 days to assess the adverse effects of chemotherapy.

The Cy3-labeled micrON™ miR-143 agomir was synthesized by RiboBio (Guangzhou, China) for histological fluorescence detection of *miR-143*.

### ^***18***^***F-FDG PET/CT analysis ***in vivo

^18^F-FDG PET/CT scan was performed on tumor-bearing mice using an Inveon small-animal PET/CT system (Siemens, Munich, Germany). Anesthesia was induced with 2% isoflurane and maintained via intraperitoneal injection of 1.25% avertin (100 μl/10 g). Mice were injected with 0.1 mL of ^18^F-FDG (130 μCi) in the tail vein and imaged with PET/CT (Siemens Inveon) under anesthesia (5-min CT scans followed by 10-min PET scans). PET images were reconstructed on an Inveon Acquisition Workplace using the 3D OSEM algorithm. The region of interest was selected on CT images and transferred to PET images. The standardized uptake value (SUV) of ^18^F-FDG was computed using the formula: SUV = [decay–corrected activity (kBq) per milliliter of tissue volume / (activity of injected ^18^F-FDG (kBq)/body mass (g)].

Under 1% isoflurane induced anesthesia, mice were performed euthanasia by intraperitoneal injection of 1% pentobarbital sodium dissolved in saline water (200 μl/10 g) until no breath, heartbeat, neural reflex and muscle tension was observed.

### Immunohistochemistry (IHC) and Fluorescence Photography

Mice were sacrificed, and tumor samples were embedded in paraffin or snap-frozen to obtain cryosections. Paraffin-embedded sections were deparaffinized, rehydrated in a graded ethanol series, and subjected to antigen retrieval. Samples were washed in phosphate-buffered saline (PBS) three times for 5 min under gentle shaking and blocked with 3% H_2_O_2_ for 25 min and 3% BSA for 30 min at room temperature. Sections were incubated with a primary antibody (anti-ENT1 and anti-HK2; Table 2) overnight at 4 °C and with an HRP-labelled secondary antibody for 1 h at room temperature. Next, peroxidase substrate DAB kit was utilized for visualization and slides were observed under a light microscope. For frozen slides from tumors injected Cy3-labeled micrON™ miR-143 agomir, Cy3 signaling was presented under a fluorescence microscope with cell nucleus dyed with DAPI.

### Statistical analysis

All data are presented as mean ± SD. Pairwise comparisons were performed using Student’s t-test and GraphPad Prism version 6.0. P-values of less than 0.05 (denoted by *, **P < 0.01, ***P < 0.001 and ****P < 0.0001) were considered statistically significant.

## Results

### Correlation of prognosis or survivability with the expression of hENT1 in TNBC patients

**Fig. 1:**
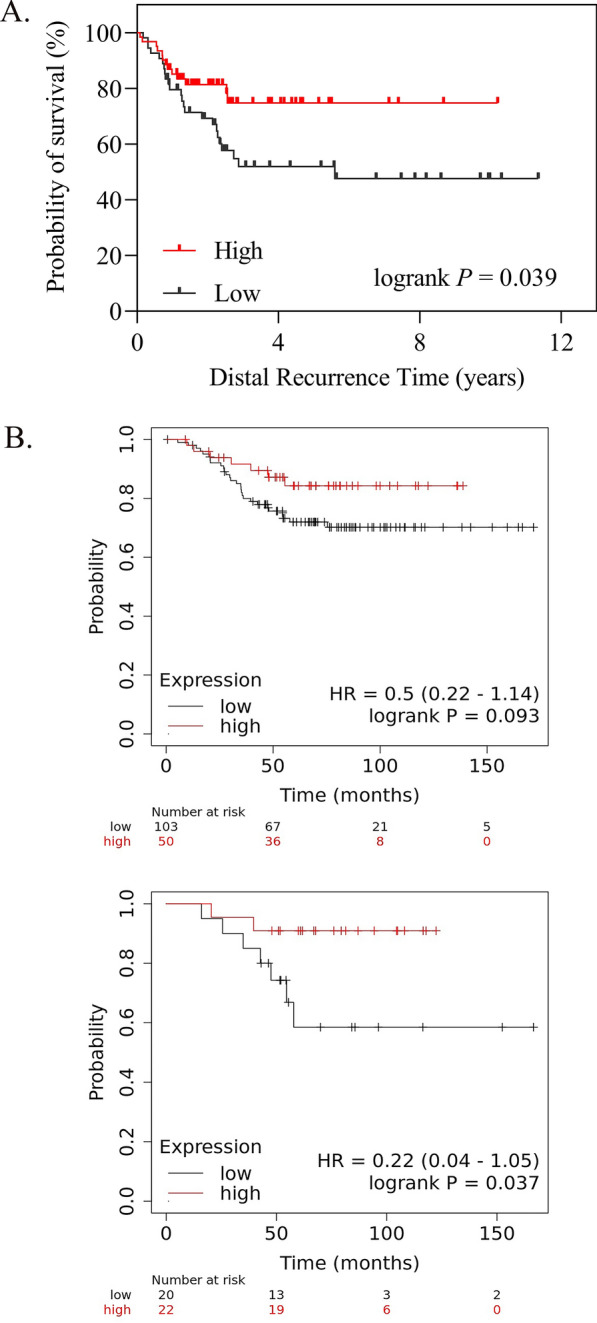
Patient survival analysis showing the correlation of prognosis or survivability with the expression of hENT1 in TNBC patients. **A** Distal recurrence survival curve in TNBC patients with high or low expression of hENT1 from GSE25066 and GSE69031 (p = 0.039). **B** Kaplan–Meier analysis showing TNBC patients (SLC29A1, 201801_s_at) with higher hENT1 expression exhibited better overall survival (p = 0.093), especially in basal-like subset TNBC patients (p = 0.037)

High expression of hENT1 showed a significant association with favorable distal recurrence free survival in TNBC patients from GSE25066 and GSE69031 (Fig. 1A, p = 0.039). From the KM Plotter cohort study, TNBC patients with higher hENT1 expression exhibited better overall survival (Fig. 1B, p = 0.093), especially in basal-like subset TNBC patients (Fig. 1B, p = 0.037), suggesting a more precise identification of the role of hENT1 in TNBC prognosis.

### *Validation of GEM-R cells *in vitro* and *in vivo

**Fig. 2:**
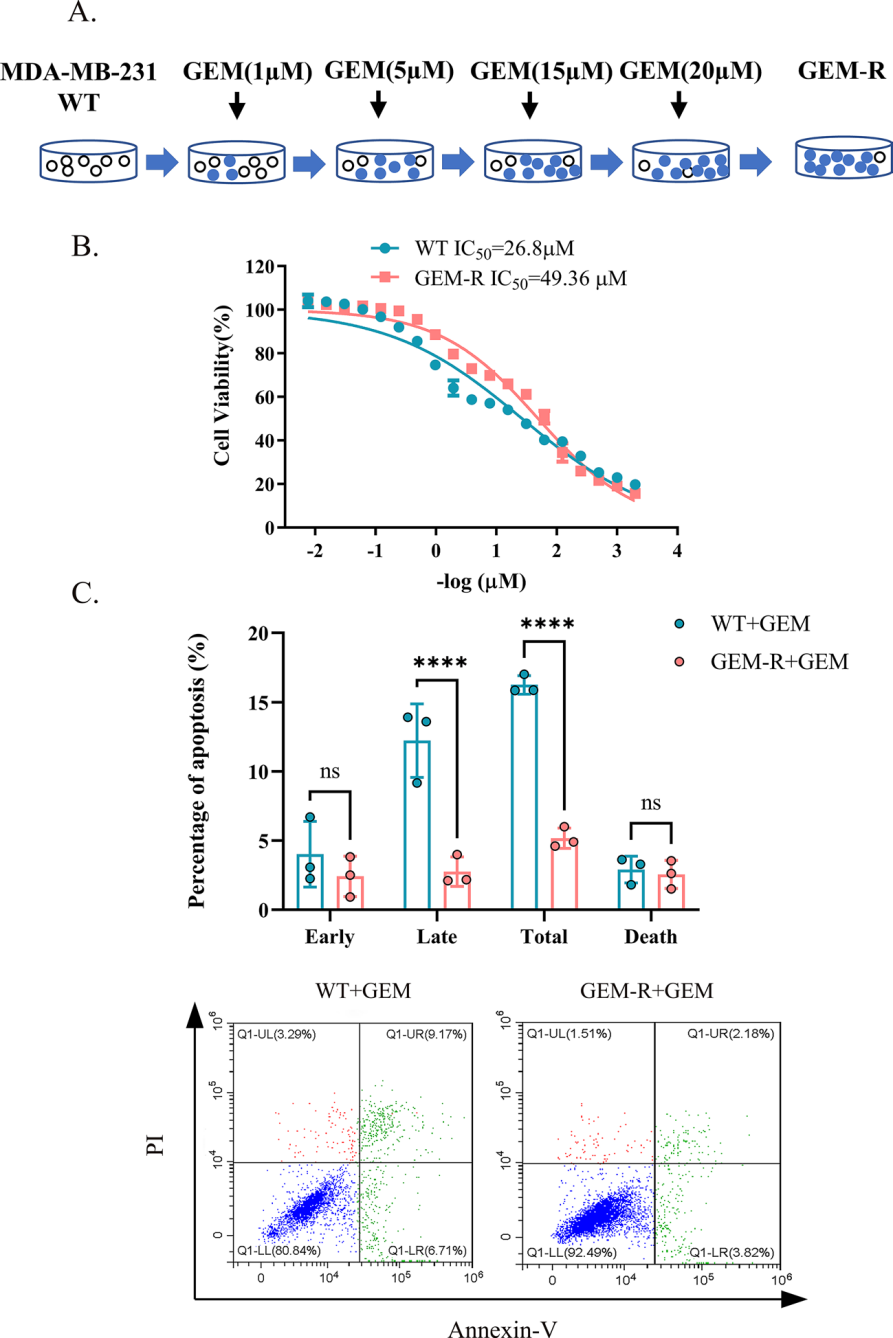
Establishment of GEM-R cells and assessment of chemosensitivity between WT and GEM-R cells in vitro. Note: GEM-R: gemcitabine-resistant MDA-MB-231 cells. Data are represented as mean ± SD. All experiments were performed in triplicate and repeated independently three times. In (C), cells were treated with 10 μM GEM for 72 h. A. Development of GEM-R cells by treating parental cells with gradient GEM (1 µM, 5 µM, 15 µM, 20 µM) after several cycles until stable proliferation. B. CCK8 assay was used to measure IC_50_ of WT cells and GEM-R cells to gradient concentration of GEM. C. Percentage of cell apoptosis of WT cells and GEM-R cells was evaluated by detection of flow cytometry with Annexin-V FITC/PI Apoptosis Detection Kit. Flow cytometric: Annexin V-/P- represented normal cells; Annexin V-/P + represented necrotic cells; Annexin V + /P + represented late apoptotic cells; Annexin V + /P- represented early apoptotic cells

**Fig. 3:**
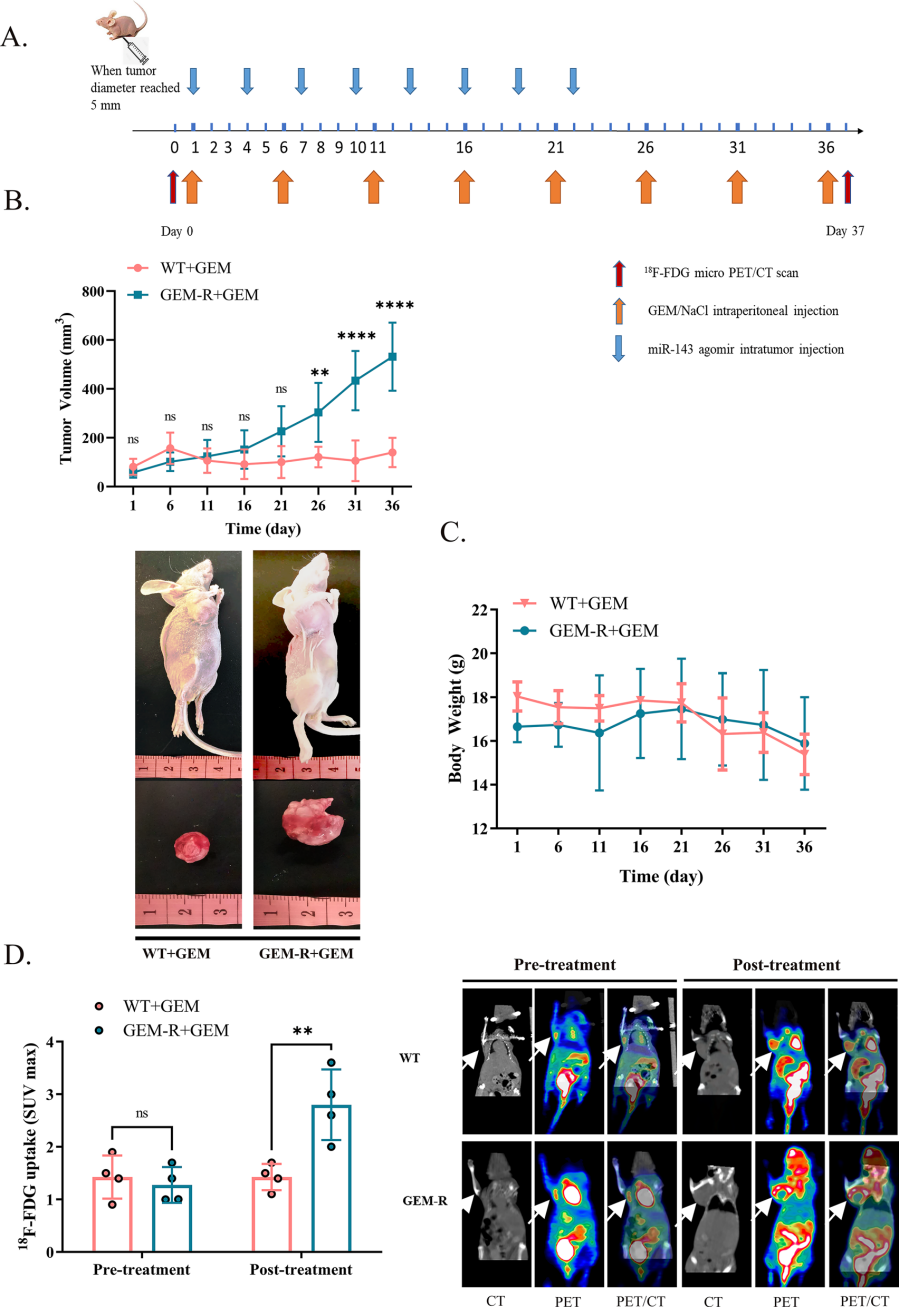
Validation of GEM-R cell lines in vivo. Note: Data are represented as mean ± SD. **A** TNBC tumor-bearing mice model and gemcitabine-resistant TNBC tumor model were established with 5-week-old BALB/c nude mice to validate chemoresistance of GEM-R cells we gained in vivo. **B** Tumor Volume growth curve was calculated to investigate the tumorigenesis and development of mice of WT and GEM-R tumor-bearing mice under GEM treatment (20 mg/kg/per every 5 days for 8 cycles). **C** Record of body weight of tumor bearing mice. **D** ^18^F-FDG microPET/CT scans were performed to analyze the maximum standardized uptake value (SUVmax) of ^18^F-FDG in chemo-therapy groups of GEM-R TNBC tumor bearing mice and TNBC tumor bearing mice before and after GEM treatment. White arrows point to tumors in mice

The viability and apoptosis of GEM-R cells were analyzed to evaluate resistance to GEM in vitro. The IC_50_ of GEM was higher in GEM-R cells than in WT cells (49.62 ± 0.34 µM vs. 26.10 ± 1.58 µM, p < 0.01) (Fig. 2B). The total rate of apoptosis was lower in GEM-R cells than in WT cells under the same treatment (16.25 ± 0.66% vs. 5.17 ± 0.73%, p < 0.0001) (Fig. 2C). In vivo, the inhibition of tumor growth by GEM was lower in GEM-R tumors than in WT tumors (p < 0.0001) (Fig. 3B). At the end of the treatment, the mean volume of GEM-R tumors was much bigger than WT tumors (Fig. 3B). There was no significant body weight loss across the groups during the treatment period (Fig. 3C). These results demonstrated that GEM-R cells are resistant to gemcitabine.

Mice of WT + GEM and GEM-R + GEM (Table 3) performed ^18^F-FDG PET/CT scan which presented the level of tumor glycolysis before and after treatment (Fig. 3D). The uptake of ^18^F-FDG after GEM treatment was significantly higher in mice bearing GEM-R tumors than in mice bearing WT tumors (mean SUVmax of 2.733 ± 0.81 and 1.425 ± 0.25, respectively; p < 0.01), demonstrating that the rate of proliferation was higher in GEM-R tumors than in WT tumors owing to the resistance to GEM and lead to higher level of glycolysis.

**Table 3:** Group allocation of WT and GEM-R tumor model mice to validate GEM resistance in vivo

Treatment	WT		GEM-R	
NaCl	Y	N	Y	N
GEM	N	Y	N	Y
n	6	6	6	6

Note: *Y* Drug applied; *N* drug not applied

### Upregulating hENT1 leads to higher apoptosis by inducing more GEM into cells

**Fig. 4:**
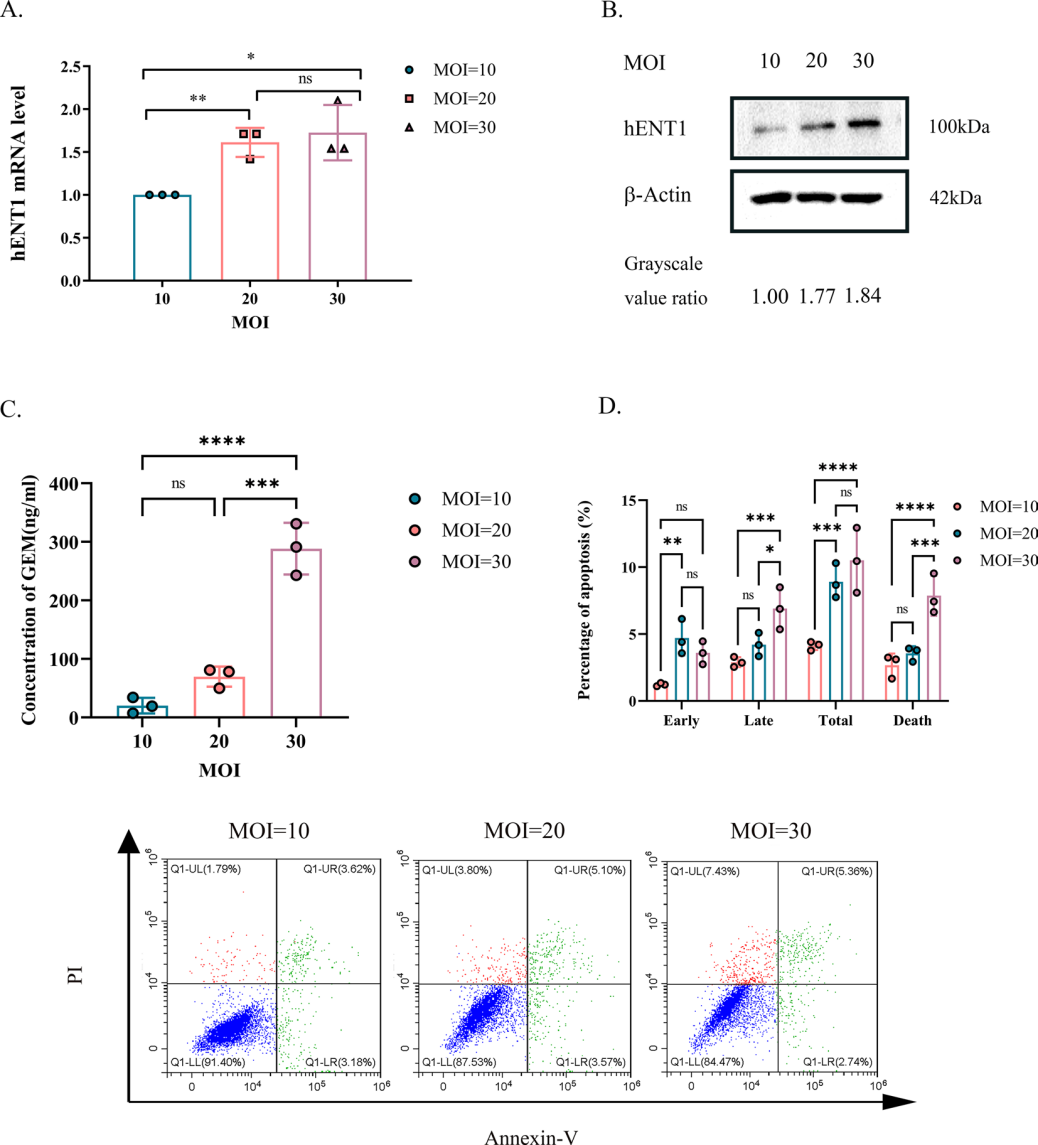
Validation of upregulating hENT1 leads to higher apoptosis by inducing more GEM into cells. Note: Data are represented as mean ± SD. All experiments were performed in triplicate and repeated independently three times. **A** RT-PCR showed mRNA expression of hENT1 increased as MOI increased. **B** Western blot showed protein expression of hENT1 increased as MOI increased. Grayscale value ratio = The area of the target protein band/The area of the endogenous reference protein band. **C** Uptake of GEM by MRM analysis in GEM-R cells with gradient hENT1 transfection. **D** Percentage of cell apoptosis of GEM-R cells transfected with gradient MOI of hENT1 was evaluated by detection of flow cytometry with Annexin-V FITC/PI Apoptosis Detection Kit. Flow cytometric: Annexin V-/P- represented normal cells; Annexin V-/P + represented necrotic cells; Annexin V + /P + represented late apoptotic cells; Annexin V + /P- represented early apoptotic cells

Lentivirus plasmids were transfected with gradient MOI (Multiplicity of Infection). The mRNA and protein expression levels of hENT1 increased with higher MOI (Fig. 4A&B). The uptake concentration of GEM in GEM-R cells increased with gradient MOI of hENT1 infection (Fig. 4C 20.15 ± 13.4 ng/ml, 69.59 ± 17.2 ng/ml and 288.31 ± 44.1 ng/ml; MOI = 10 vs. 20, p < 0.001, MOI = 10 vs. 30, p < 0.0001). Higher uptake of GEM lead to higher rate of apoptosis (Fig. 4D, 4.12 ± 0.32%, 8.91 ± 1.3%, 10.50 ± 2.42%; MOI = 10 vs. 20, p < 0.0001; MOI = 10 vs. MOI = 30, P < 0.0001).

### Overexpression of hENT1 reverses GEM resistance in GEM-R cells and in vivo

**Fig. 5:**
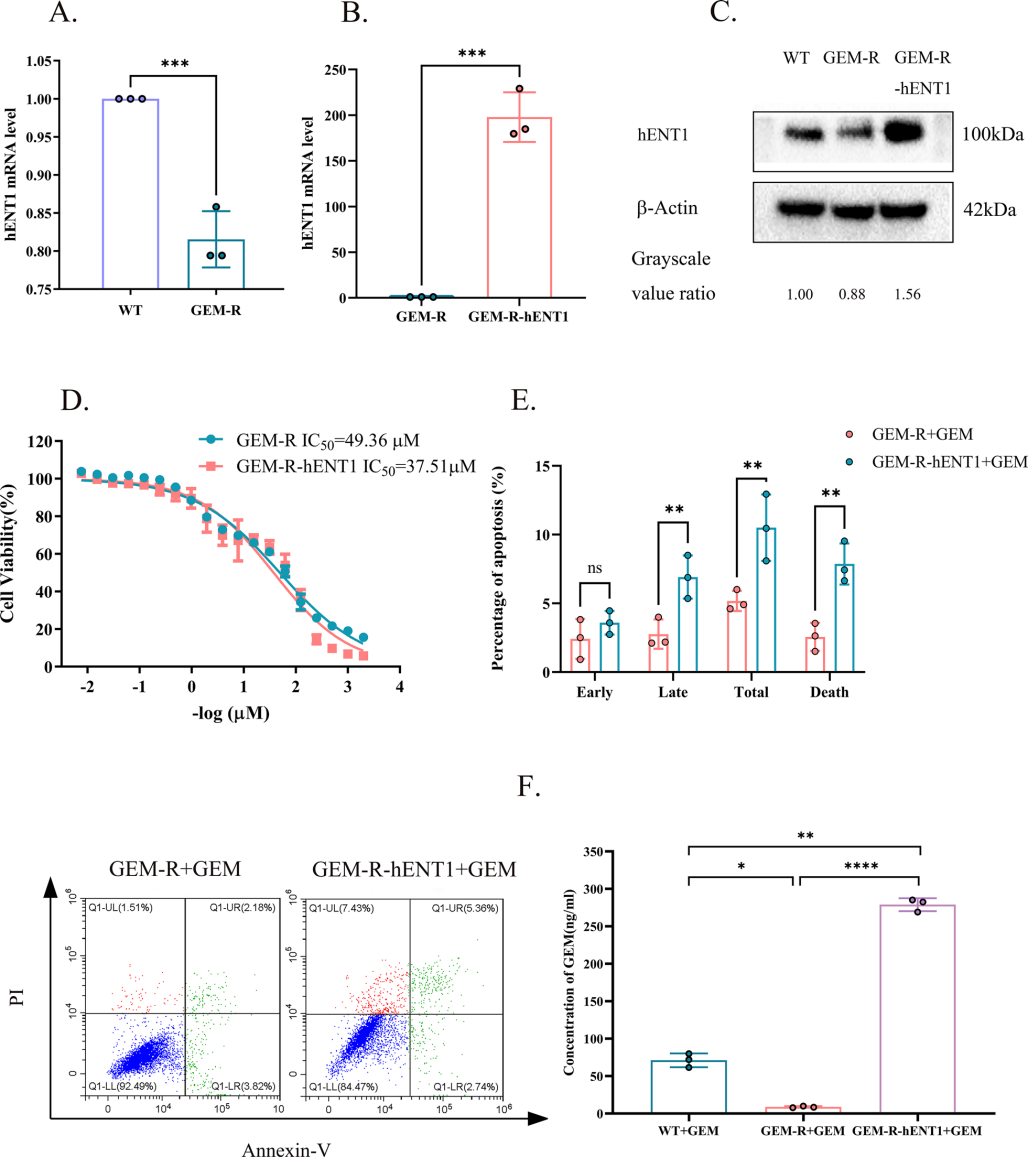
Overexpression of hENT1 in GEM-R cells and its reversal of chemoresistance in vitro. Note: Data are represented as mean ± SD. All experiments were performed in triplicate and repeated independently three times. **A** RT-PCR showed expression of hENT1 in mRNA level between WT and GEM-R cells. **B** RT-PCR showed overexpression of hENT1 in mRNA level in GEM-R-hENT1 cells compared with GEM-R cells. **C** Western blot showed expression of hENT1 in protein level of WT, GEM-R cells and GEM-R-hENT1 cells. Grayscale value ratio = The area of the target protein band/The area of the endogenous reference protein band. **D** CCK8 assay was used to evaluate IC_50_ of GEM-R cells and GEM-R- hENT1 cells to gradient concentration of GEM. **E** Percentage of cell apoptosis of GEM-R cells and GEM-R- hENT1 cells was evaluated by detection of flow cytometry with Annexin-V FITC/PI Apoptosis Detection Kit. **F** Uptake of GEM in WT, GEM-R and GEM-R- hENT1 cells by MRM analysis. Flow cytometric: Annexin V−/P− represented normal cells; Annexin V−/P + represented necrotic cells; Annexin V + /P + represented late apoptotic cells; Annexin V + /P- represented early apoptotic cells

The mRNA (Fig. 5A) and protein expression (Fig. 5C) levels of hENT1 were lower in GEM-R cells than in WT cells. We overexpressed hENT1 in GEM-R cells to confirm the hypothesis that hENT1 overexpression reverses GEM resistance. The mRNA (Fig. 5B) and protein expression (Fig. 5C) levels of hENT1 were higher in GEM-R cells with exogenous expression of the hENT1 gene (GEM-R-hENT1 cells) than in GEM-R cells. The viability and apoptosis of GEM-R and GEM-R-hENT1 cells after GEM treatment were analyzed. The viability of GEM-R-hENT1 cells was lower than that of GEM-R cells under the same treatment (IC_50_ of 36.90 ± 0.80 µM and 49.62 ± 0.34 µM, respectively, p < 0.01) (Fig. 5D). In addition, the rate of apoptosis was higher in GEM-R-hENT1 cells than in GEM-R cells (10.50 ± 2.42% vs. 5.17 ± 0.73%, p < 0.01) (Fig. 5E). The uptake concentration of GEM in GEM-R-hENT1 cells (278.9 ± 8.61 ng/ml) was significantly higher than GEM-R cells (8.7 ± 1.13 ng/ml, p < 0.0001) and WT cells (71.2 ± 9.20 ng/ml, p < 0.01) (Fig. 5F). These findings indicated that hENT1 overexpression increased the influx of GEM, and thus reversed GEM resistance in GEM-R cells.

**Fig. 6:**
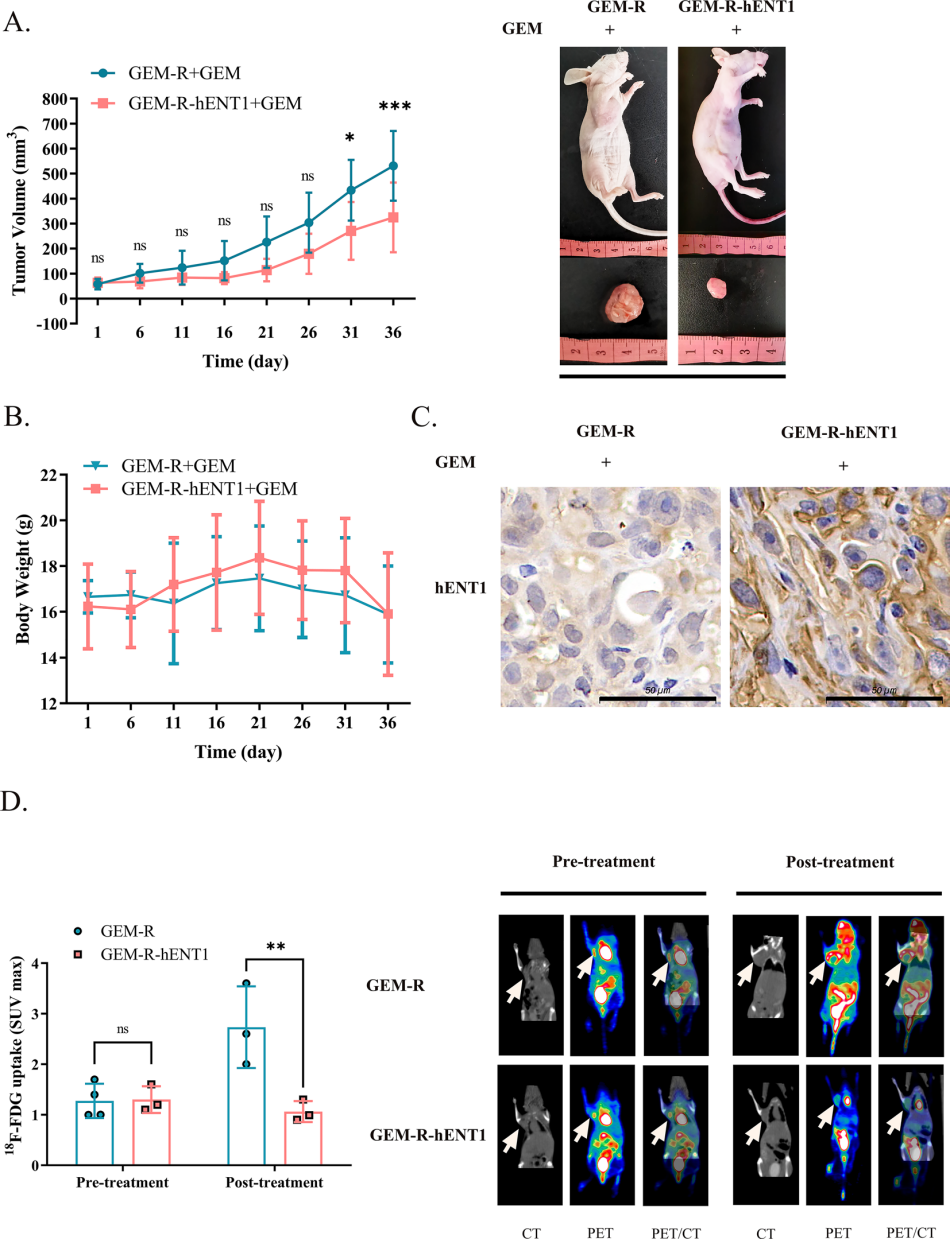
Verification of therapeutic effect of hENT1 overexpression in vivo. Note: Data are represented as mean ± SD. **A** Tumor Volume growth curve was calculated to investigate the tumorigenesis and development of mice of each group. **B** Record of body weight of tumor bearing mice. **C** Photographs (365 ×) of immunohistochemistry staining on hENT1 of tumor sample. **D** ^18^F-FDG microPET/CT scans were performed to analyze the maximum standardized uptake value (SUVmax) of ^18^F-FDG in GEM-R TNBC tumor bearing mice and GEM-R-hENT1 tumor bearing mice before and after GEM treatment. White arrows point to tumors in mice

In vivo (Table 4), the tumor volume of mice bearing GEM-R-hENT1 tumors grew slower (Day 31, p < 0.05; Day 36, p < 0.001) than that of mice bearing GEM-R tumors after GEM treatment (Fig. 6A). No significant difference in body weight loss was observed across the groups during treatment (Fig. 6B). Immunohistochemistry showed that GEM-R-hENT1 tumors had higher hENT1 expression levels than GEM-R tumors (Fig. 6C). In addition, GEM decreased the uptake of ^18^F-FDG in the GEM-R-hENT1 group compared with the GEM-R group (mean SUVmax of 1.063 ± 0.21 vs. 2.733 ± 0.81, p < 0.01), suggesting that hENT1 overexpression reversed cancer drug resistance in vivo (Fig. 6D).

**Table 4:** Group allocation of treatment experiments in an animal model. Group allocation of GEM-R and GEM-R-hENT1 tumor model mice to validate hENT1 therapy in vivo

Treatment	GEM-R		GEM-R-hENT1	
GEM	N	Y	N	Y
n	6	6	6	6

Note: Y: Drug applied; N: Drug not applied

### MiR-143 suppressed glycolysis in GEM-R cells

**Fig. 7:**
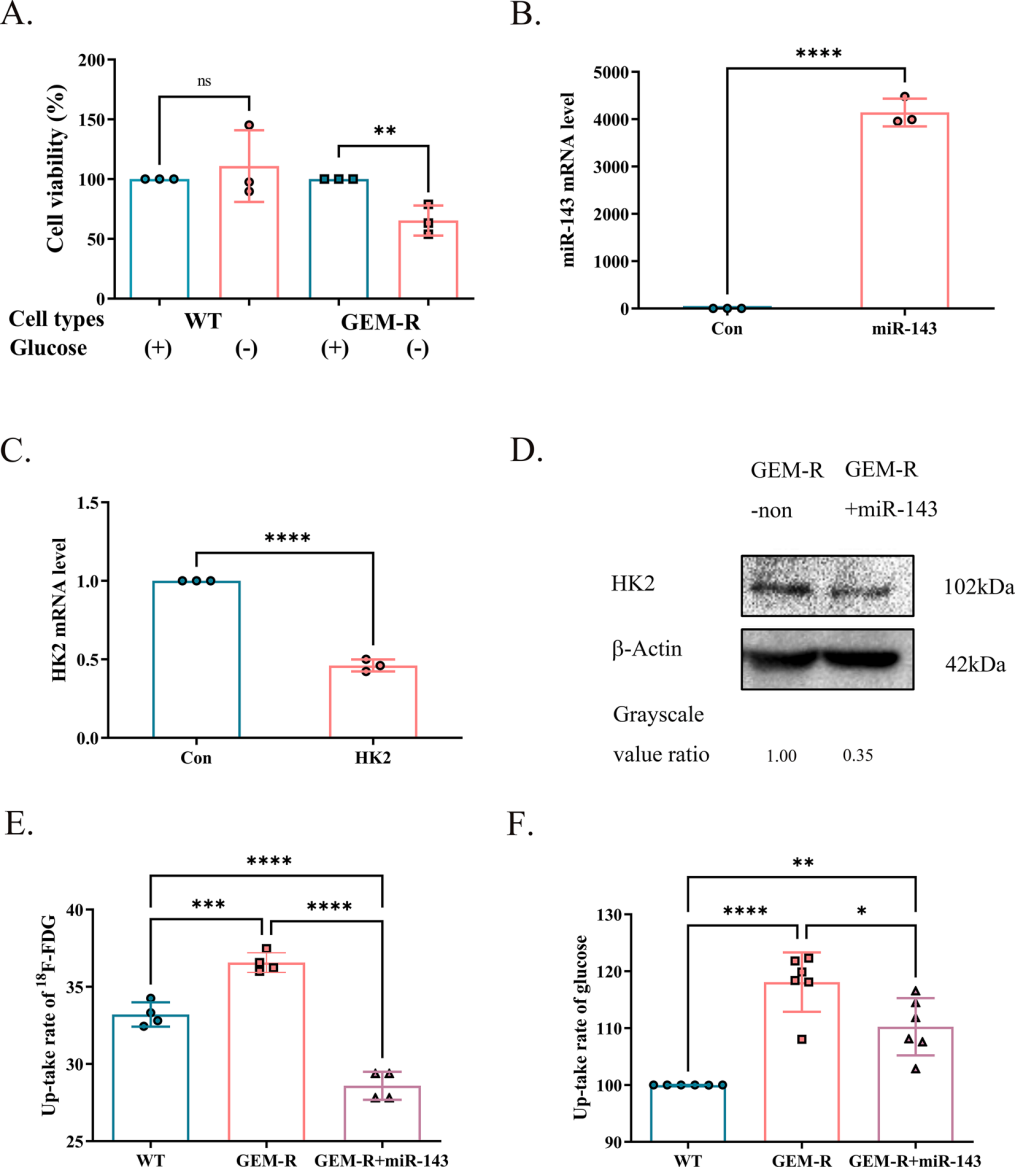
Glycolysis in WT and GEM-R cells and effect of *miR-143* on gem-resistance in vitro. Note: Data are represented as mean ± SD. All experiments were performed in triplicate and repeated independently three times. **A** CCK8 assays were utilized to evaluate cell viability of GEM-R cells and their parental ones with or without glucose intervention. **B** RT-PCR showed higher *miR-143* level in *miR-143* induced GEM-R cells compared with control group. C&D. RT-PCR and Western blot were performed to measure HK2 expression from mRNA and protein levels. Grayscale value ratio = The area of the target protein band/The area of the endogenous reference protein band. E. PET/CT scan was performed to assess the cellular uptake of ^18^F-FDG in WT cells, GEM-R cells and GEM-R cells transfected with *miR-143*. F. Glucose assay kit was used to test glucose consumption among three types of cells

The result of GEM-R cells showing lower cell viability when cultured in the absence of glucose revealed that GEM-R cells were glucose-dependent and exhibited more active glycolysis (p < 0.01, Fig. 7A). We previously demonstrated that *miR-143* mimic suppressed glycolysis in MDA-MB-231 cells by downregulating HK2 [47]. In the present study, GEM-R cells transfected with *miR-143* mimic (p < 0.0001, Fig. 7B) also showed decreased mRNA (p < 0.0001) and protein expression of HK2 (Fig. 7C and D) as well as lower ^18^F-FDG uptake (p < 0.0001) and glucose metabolic rate (p < 0.001) than GEM-R cells without miR-143 treatment (Fig. 7E and F), indicating that *miR-143* inhibited glycolysis in GEM-R cells by downregulating HK2.

### MiR-143 enhanced the effect of reversing GEM resistance in GEM-R-hENT1 cells and in vivo

**Fig. 8:**
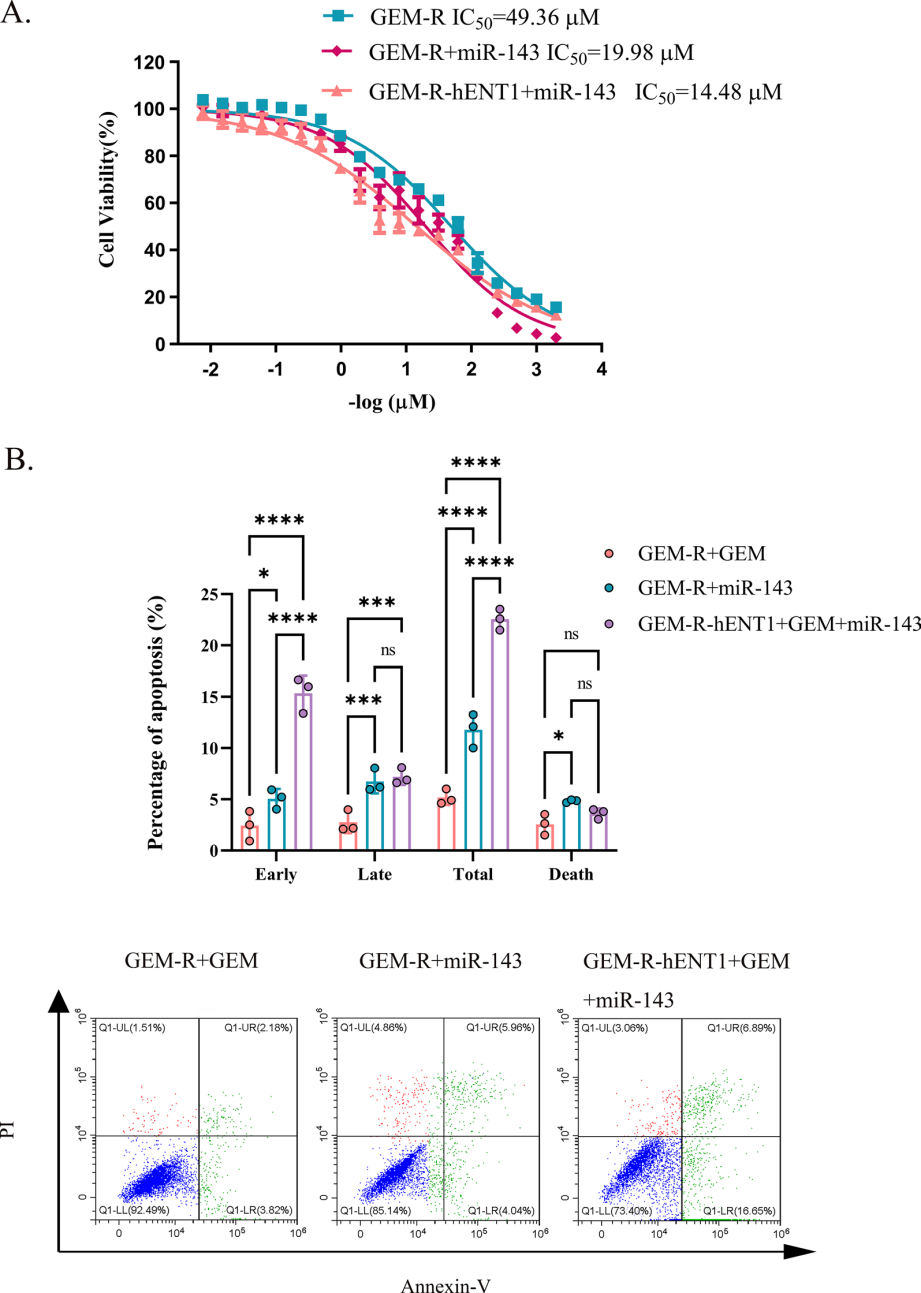
Efficacy of hENT1 adjuvant with *miR-143* on gem-resistance in vitro. Note: Data are represented as mean ± SD. All experiments were performed in triplicate and repeated independently three times. **A** CCK8 assay was used to evaluated IC_50_ of GEM-R cells, GEM-R cells transfected with *miR-143* and GEM-R-hENT1 cells induced with *miR-143* when treated with gradient concentration of GEM. **B** Percentage of cell apoptosis of GEM-R cells, GEM-R cells transfected with *miR-143* and GEM-R-hENT1 cells induced with *miR-143* was evaluated by detection of flow cytometry with Annexin-V FITC/PI Apoptosis Detection Kit. Flow cytometric: Annexin V-/P- represented normal cells; Annexin V-/P + represented necrotic cells; Annexin V + /P + represented late apoptotic cells; Annexin V + /P- represented early apoptotic cells

In vitro, the viability of *miR-143*-treated GEM-R-hENT1 cells was lower than that of *miR-143*-treated GEM-R and control GEM-R cells (IC_50_ of 14.71 ± 0.57 µM, 19.30 ± 0.59 µM, and 49.62 ± 0.34 µM, respectively; p < 0.0001, p < 0.0001 compared with control GEM-R cells) after GEM treatment (Fig. 8A). Similarly, the rate of apoptosis was higher in the first group than in the latter two groups (22.54 ± 1.03%, 11.79 ± 1.66% and 5.17 ± 0.73%, respectively; p < 0.0001, p < 0.0001; Fig. 8B).

**Fig. 9:**
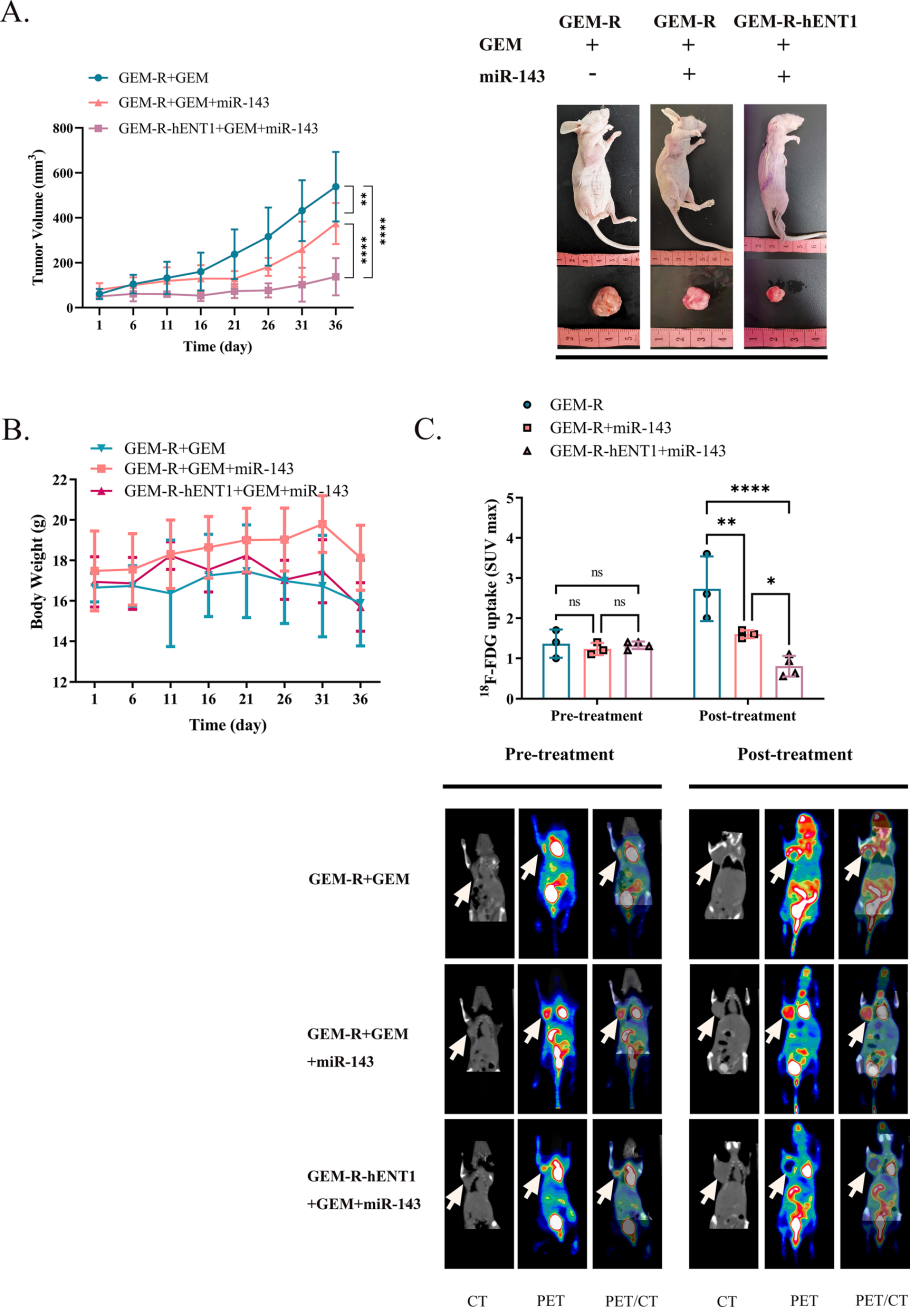
Efficacy of hENT1 adjuvant with *miR-143* on gem-resistance in vivo. Note: Data are represented as mean ± SD. **A** Tumor Volume growth curve was calculated to investigate the tumorigenesis and development of mice of each group. **B** Record of body weight of tumor bearing mice. **C** ^18^F-FDG microPET/CT scans were performed to analyze the maximum standardized uptake value (SUVmax) of ^18^F-FDG in GEM-R TNBC tumor bearing mice and GEM-R-hENT1 tumor bearing mice before and after GEM treatment or GEM-miR-143 dual treatment

**Fig. 10:**
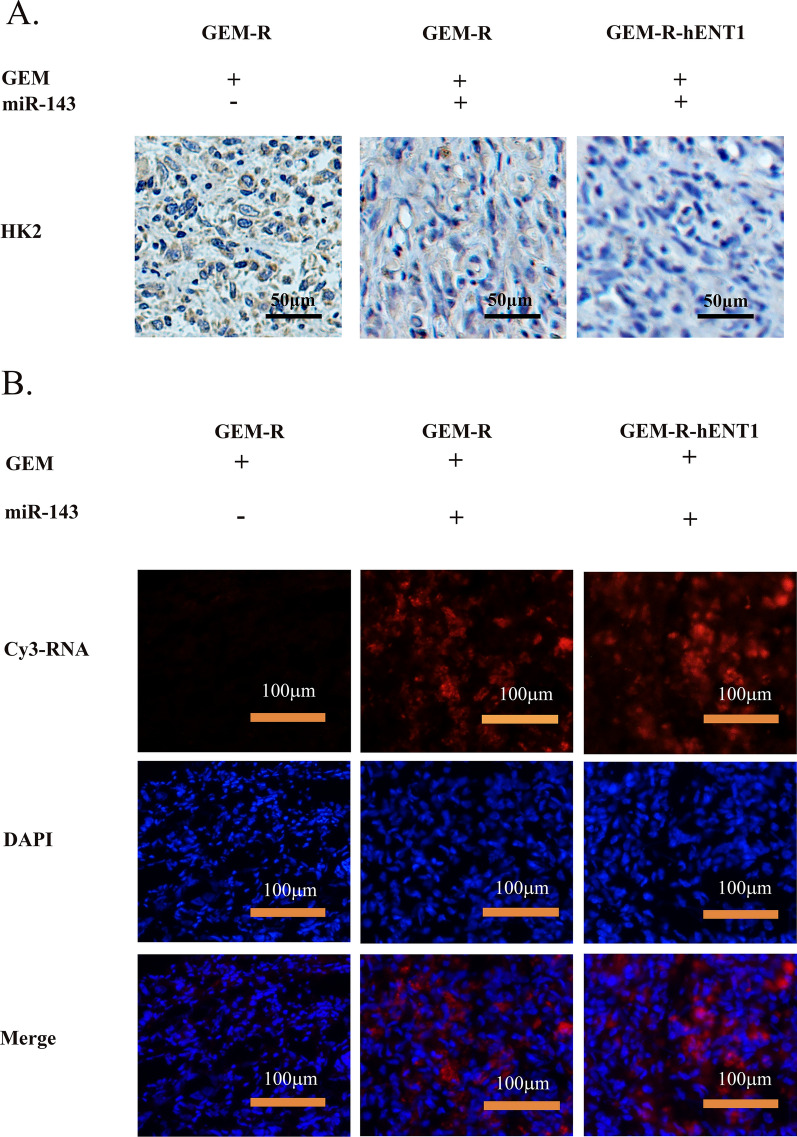
Tumor sample detection of mice model of hENT1 adjuvant with *miR-143* on gem-resistance. **A** Photographs (200 ×) of immunohistochemistry staining on HK2 of tumor samples. **B** Fluorescence photography (200 ×) was applied to detect Cy3 signals to support the administration of *miR-143* agomir in the tumors

In vivo (Table 5), tumor growth inhibition was higher in *miR-143* + GEM-treated mice bearing GEM-R-hENT1 tumors than in *miR-143* + GEM-treated mice bearing GEM-R tumors (p < 0.01) and control GEM-R tumors (p < 0.0001) after GEM treatment (Fig. 9A). In addition, there was no statistically significant difference in body weight across the groups (Fig. 9B). Moreover, ^18^F-FDG uptake was significantly lower in the group of GEM-R-hENT1 + GEM + *miR-143* (p < 0.0001, Fig. 9C) with lower HK2 expression on immunohistochemical staining compared with the other two groups (Fig. 10A). Fluorescence imaging dentified Cy3-labeled *miR-143* agomir in the tumor tissues of the *miR-143*-treated groups (Fig. 10B). These results indicated that *miR-143* inhibited glycolysis by regulating HK2 expression and reduced cancer drug resistance in a mouse xenograft tumor model, enhancing the effect of hENT1-induced reversal of GEM resistance.

**Table 5:** Group allocation of treatment experiments in an animal model. Group allocation of GEM-R and GEM-R-hENT1 tumor model mice to validate dual-gene therapy in vivo

Treatment	GEM-R	GEM-R-hENT1	
GEM	Y	Y	Y
MiR-143	N	N	Y
n	6	6	6

Y: Drug applied; N: Drug not applied

## Discussion

Gemcitabine resistance is a critical problem in the treatment of TNBC. In this study, we designed a combination therapy involving hENT1 overexpression and *miR-143* administration, and investigated its efficacy in reversing GEM resistance in vitro and in an animal model of TNBC.

The nucleoside transporter hENT1 mediates GEM uptake in vitro and in vivo [25, 53, 54] and GEM resistance [27, 55, 56]. In this respect, Santini D et al*.* have shown that hENT1 expression is a prognostic marker in adjuvant therapy with GEM in patients with radically resected gastric cancer [57]. Marcé et al*.* [58] observed that the expression of hENT1 was closely correlated with GEM uptake and toxicity in mantle cell lymphoma. Our data suggested high expression of hENT1 generally predicted favorable prognosis in TNBC. It is worth noting that hENT1 was significantly associated with increased overall survival in basal-like 1 subset in TNBC patients. Given that TNBC is particularly heterogenous in breast cancer, further study for hENT1 expression in stratification of TNBC patients were required.

We found that upregulating the expression of hENT1 reversed GEM resistance in TNBC by mediating GEM influx into tumor cells [25] and inhibited tumor growth. However, the effect on reversing GEM resistance of increase in hENT1 levels and GEM influx in GEM-R-hENT1 cells was limited and slow. There wasn’t obvious inhibition of tumor growth until Day 31 during the late stages in the treatment and there were large individual differences in tumor growth inhibition rate, possibly because of high redundancy in transport which meant only part of the hENT1 protein we upregulated played a role [45].

Furthermore, glycolysis was found to be higher in GEM-R cells than in WT cells in our study, suggesting that GEM resistance was accompanied by increased glycolysis [59]. Under aerobic conditions, tumor cells have higher glycolytic activity and reduced oxidative phosphorylation (Warburg effect) [60]. There were three reasons: to prepare glycolytic intermediates into the pentose phosphate pathway (PPP) for the rapid proliferation of tumor cells [61], to faster content a sudden increase of energy demand [62, 63], to protect tumor cells against oxidative stress [61]. Icard et al*.* reported [64] that the Warburg effect determines the aggressiveness and drug resistance of cancer cells partly by accelerating epigenetic and genetic changes. Enhanced glycolysis promotes drug resistance in pancreatic cancer [65], glioblastoma [66], and breast cancer [67]. Dai et al*.* proved that pancreatic cancer cell survival and tolerance to GEM were reduced by 2-deoxy-D-glucose-induced inhibition of glycolysis [65]. Moreover, TNBC exhibits abnormally increased glucose uptake and depends more on glycolysis than other breast cancer subtypes [68, 69]. Its possible mechanisms included a) the downregulation of transporters such as hENT1 in GEM-R cells decreased adenosine entry into cells, and adenosine accumulation in the extracellular environment stimulated increase of glycolysis in tumor cells to generate energy through the AMPK pathway [35, 36], b) our previous study [51] indicated that c-Myc and HIF-1α, two key regulators of glucose transport and metabolism, were more highly expressed in GEM-resistant pancreatic cancer cells than GEM-sensitive cells, confirming the crosstalk between glycolysis and chemoresistance. Therefore, the inhibition of glycolysis can potentially enhance the therapeutic effect of hENT1 treatment in GEM-R cells.

As a glycolysis inhibitor, *miR-143* inhibited glycolysis in breast cancer cells [42] by downregulating the expression of HK2, which is a pivotal regulator of aerobic glycolysis and tumor growth [70, 71], and the systemic delivery of *miR-143* agomir inhibited tumor growth [47]. Li et al*.* showed that *miR-143* as significantly decreased in TNBC, and *miR-143* overexpression could reduce the proliferation of TNBC cells by regulating the LIMK1/CFL1 pathway [72]. In the present study, we demonstrated that *miR-143* reduced drug resistance in GEM-R cells by suppressing glycolysis and enhanced the effect of GEM reversal of hENT1 overexpression. Further combination therapy involving hENT1 overexpression and *miR-143* administration achieved a higher tumor growth inhibition rate than hENT1 or *miR-143* treatment alone, which can potentially be used to reverse drug resistance in TNBC. Without GEM resistance, GEM functions as cytidine analog and strongly induces apoptosis [73, 74] in breast cancer in vitro and in vivo [75].

There are some limitations in our study. First, exogenously upregulating the expression of hENT1 through transfection of recombinant lentivirus may have potential biosafety risks. Second, *miR-143* was administered by intratumor injection; therefore, additional studies are necessary to assess whether *miR-143* mimic can produce a considerable inhibitory effect on GEM-resistant TNBC tumors through systemic administration. Third, the detail mechanism by which *miR-143* reverses GEM resistance needs further exploration. Fourth, the regulation of drug efflux by ATP-binding cassette transporters, which [76] have also been shown to reverse drug resistance in breast cancer cells, was not investigated in the present study.

## Conclusions

Increased hENT1-mediated GEM influx into GEM-R cells reversed GEM resistance and *miR-143*-mediated inhibition of glycolysis could significantly enhance this effect. Thus, hENT1 combined with *miR-143* is a promising strategy for treating GEM-resistant TNBC.

## Data Availability

The datasets used during the study are available from the corresponding author on reasonable request.

## Abbreviations

TNBC: Triple-negative breast cancer
WT: MDA-MB-231
GEM-R: Gemcitabine-resistant MDA-MB-231
GEM-R-hENT1: GEM-R cells with exogenous expression of the hENT1 gene
